# Molecular docking and pharmacokinetic evaluation of natural compounds as targeted inhibitors against Crz1 protein in Rhizoctonia solani

**DOI:** 10.6026/97320630015277

**Published:** 2019-04-15

**Authors:** Ajit Malik, Sarah Afaq, Basiouny El Gamal, Mohamed Abd Ellatif, Waleed N Hassan, Ayed Dera, Rana Noor, Mohammed Tarique

**Affiliations:** 1 Department of Clinical Biochemistry, College of Medicine, King Khalid University, Abha, Saudi Arabia; 2 Center for InterdisciplinaryResearch in Basic Sciences, Jamia Millia Islamia, Jamia Nagar, New Delhi-110025, India; 3 Department of Medical Biochemistry,Faculty of Medicine, Mansoura University, Mansoura, Egypt; 4 Departments of Clinical Laboratory Science, College of Applied MedicalScience, King Khalid University, Abha, Saudi Arabia; 5 5Department of Biochemistry, Faculty of Dentistry, Jamia Millia Islamia, New Delhi-110025, India

**Keywords:** QSAR, QSTR, Crz1, pharmacokinetic, chickpea

## Abstract

Crz1p regulates Calcineurin, a serine-threonine-specific protein phosphatase, in Rhizoctonia solani. It has attracted consideration as a novel target of antifungal therapy based on studies in numerous pathogenic fungi, including, Cryptococcus neoformans, Candida albicans and Aspergillus fumigatus. To investigate whether Calcineurin can be a useful target for the treatment of Crz1 protein in R. solani causing wet root rot in Chickpea. The work presented here reports the in-silico studies of Crz1 protein against natural compounds. This study Comprises of quantitative structure-toxicity relationship (QSTR) and quantitative structure-activity relationship (QSAR). All compounds showed high binding energy for Crz1 protein through molecular docking. Further, a pharmacokinetic study revealed that these compounds had minimal side effects. Biological activity spectrum prediction of these compounds showed potential antifungal properties by showing significant interaction with Crz1. Hence, these compounds can be used for the prevention and treatment of wet root rot in Chickpea.

## Background

Calcineurin is essential for cell viability under specific environmental conditions 1. Calcineurin is a heterodimer of a catalytic subunit (genes of CNA1 and CNA2) and a regulatory subunit (CNB1) 2. Calcineurin deficient cells (CNA1, CNA2, and CNB1) failed to grow in the presence of Na+/Li+, Mn2+, alkaline pH, and lose viability in prolonged incubation with mating pheromone 3. All of these environments, as well as the addition of Ca2+ to the growth medium, encourage Ca2+/ calcineurindependent gene expression 3, 4. A 24 bp region of the FKS2 promoter, the CDRE, is enough to direct Ca2+-induced gene expression, and this transcription requires both calcineurin and the Crz1p transcription factor 4, 5. Thus, many of the physiological functions of calcineurin in Rhizoctonia solani are mediated by its regulation of Crz1p. Calcineurin has attracted contemplation as a novel target of antifungal therapy based on previous studies in various fungi, e.g. C. neoformans, C. albicans and A. fumigates 6-10. To date, very little is known about the calcineurin pathway in Rhizoctonia solani.R. solani is a universal soil borne necrotroph that exacts harm on an extensive variety of economically important crops like pepper, tomato, eggplant and chickpea 11-13 . The fungus can damage organic substance in the soil as a saprobe and is a vital evolutionary connection between beneficial and plant disease-causing fungi 12, 14. Root rot, seedling blight, and hypocotyl rots are characteristic symptoms detected when vulnerable genotypes are implanted on high-risk fields and in conditions encouraging for disease development 15. Unfortunately, our understanding of the mechanisms promoting infection and other aspects of hostpathogen interaction is limited, impeding the progress of resistant genotypes. The transcription factor Crz1 is a downstream effector of calcineurin and is involved in azole tolerance in C. albicans 6; however, a Crz1 homolog in R. solani has yet to be characterized. Therefore, it is of interest to study natural compounds as targeted inhibitors against Crz1 protein in R. solani causing wet root rot in Chickpea.

## Methodology

### 

**Figure 1:**
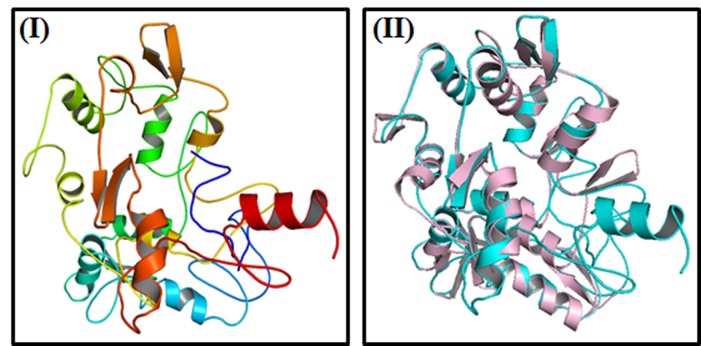
(I) Three-dimensional structure of Solano Crz1 protein predicted by I-TASSER. (II) Alignment of query protein (cyan) with structural analog (light pink) 5v3jE in PDB library.

Three dimensional structure prediction byI-TASSER: Schematic diagram of the molecular docking studies is illustrated in Figure 1. The sequence of 382 amino acids of Crz1 protein in R.solani was retrieved from the Swiss Prot database in FASTA format (Figure 3A). The three-dimensional model was generated using the I-TASSER server which makes a 3D model of query sequence by multiple threading alignments and iterative structural assembly simulation 16, 17. The reasons behind the selection of this server were its availability, composite approach of modeling and performance in CASP competition. I-TASSER methodology includes general steps of threading, structural assembly, model selection, refinement, and structure-based functional annotation 18. Then, the query sequence was threaded through the representative PDB structure library using LOMETS 19. Then, the query sequence was threaded through the representative PDB structure library using LOMETS 20. The quality of template alignment was checked by Z-score and the best one used for further consideration. In the next step, the continuous fragments in threading arrangements were excised to form an assembled structure model of aligned regions. The modeling accuracy of unaligned areas is generally low while threading aligned regions have high efficiency, so these template fragments keep rigid in the simulation process to the obtained high-resolution structure.The replica exchange Monte Carlo simulation technique was used for fragment assembly 21. The simulation includes Ca/side chain correlation, H-bonds, hydrophobicity, spatial restraints from threading templates 20, and sequence-based contact predictions from SVMSEQ 22. The conformations generated during refinement simulation process were clustered by SPICKER 23, and the average of three-dimensional coordinates of all the assembled structure was calculated to obtained cluster centroids. In the refinement process, the selected cluster centroids were again used to perform further fragment assembly simulation which helps to remove steric clashes and to refine the global topology of the cluster centroids. The PDB structures, structurally closed to the cluster centroids, were identified by TM-align 24. The final structural models were generated by REMO 25in which cluster centroids of second-round simulation used as input. In the last step, the function of three-dimensional models of query protein was predicted by matching the proteins of known structure and function in PDB. The functional analogs were ranked by TM-score, RMSD, sequence identity, and coverage of the structure alignment. The quality of the predicted model was determined by C-score (confidence score) which is ranged as -5 to 2. It depends on the quality of threading alignment and the convergence of structural assembly refinement simulations.

### Validation of predicted model:

The Ramachandaran plot further validated the confirmation of the best model of Crz1 predicted by I-TASSER. The evidence of the expected model was calculated by analyzing the phi (F) and psi (?) torsion angles using PROCHECK online server. The Ramachandaran plot obtained from PROCHECK describes a good quality model which has over 90% residues in the most favored region. The assessment of the model was also confirmed by MolProbity online server 26, 27. Prediction of active site of model: The active sites of Crz1 target proteins were identified by using computed atlas of surface topography of proteins (CASTp) server (http://cast.engr.uic.edu). It defines all the possible pockets in the protein structure. It measures the area and volume of each pocket and cavity analytically, both in solvent accessible surface and molecular surface^28^. Here, we input the Crz1 target protein for predicting the ligand binding sites, and the CASTp server predicts the amino acids crucial for binding interactions for docking studies. Preparation of Ligands and Protein Molecule: The 3D structure of Crz1 was predicted by I-TASSER. Then, the hydrogen atoms having polar nature were added, the residue structures having lower occupancy were deleted, and the incomplete side chains were later replaced by using Auto Dock Tools (ADT) version 1.5.6 from the Scripps Research Institute. Further, to each atom having Gasteiger charges were added and the non-polar hydrogen atoms were merged to the protein structure. After that, the structures constructed were saved in PDBQT file format, for further analysis in ADT 29. The next crucial step was the preparation of Crz1 known inhibitors, and 532 natural compounds as a Molfile achieved using the PubChem Database. Known inhibitors of Crz1 include Fludioxonil 15. The Mol file of the ligand was then generated and converted into PDBQT file by a process like detect root, chose the torsion and set the number of torsion by using ADT 29.

### Receptor Grid Formation:

Grid pre-calculates grid maps of binding energies for numerous atom types, such as hydrogen bonding oxygen, aromatic carbons, and aliphatic carbons with a macromolecule such as an RNA/ DNA, protein before docking 30. These grid maps are then used by AutoDock 4.2 docking calculations to define the total binding energy for a ligand with a macromolecule 29. Grid mapping calculates the crucial coordinates over the atomic protein data and assigns the coordinates of the Crz1 for docking. Also, grid mapping affords a proper surface topology for the atoms of compounds for interaction with the Crz1 active site. Grid mapping is a requirement to direct Crz1 inhibitor and 532 natural compounds to look for their region of the strong affinity of the Crz1 active site. The grid dimensions for Crz1 protein was 60 x 62 x 60 grid points with spacing 0. 463 Å between the grid points but centered on the ligand for protein (72.94, 86.112 and 72.671 coordinates). The grid was created for the search of promising interaction and best support during the docking which represents an orientation, position, confirmation about the receptors 31. 

### Molecular docking:

AutoDock 4.2 with standard protocol was used to dock the Crz1 inhibitor and 532 natural compounds into the active site of Crz1 29, 32. The energy calculations were done using the Lamarckian genetic algorithm (LGA). The conformations with the most favorable free binding energy were selected for analyzing the interactions between the target receptor and ligands by PyMOL^29^.

### In silico pharmacokinetics analysis:

#### ADMET Predictions:

Discovery studio 3.5 (Accelrys San Diego, USA) was used to generate ADMET values. The Absorption, Distribution, Metabolism, Excretion, and Toxicity commonly abbreviated as ADMET properties are considered before designing a drug as these properties play a vital role in clinical phases. Administration of these properties before drug designing leads to cost savings in drug design 33. These studies resulted in the identification of lead natural compounds. TOPKAT administers the after-effects of drug intake. It assesses the toxicological endpoints by Quantitative Structure Toxicity Relationship (QSTR). Ames mutagen predication, Ames probability, Ames enrichment and weight of evidence are tested by toxicity profile of the compounds.

#### Measuring drug-likeness:

The drug-like properties of compounds were analyzed by the online tool Molinspiration server (http://www.molins piration.com). Molinspiration support for calculation of critical molecular properties is based on Lipinski Rules of five such as (molecular weight, number of hydrogen bond donors and acceptors) 34.This server also predicts bioactivity score for the most important therapeutic targets like GPCR receptors, kinase inhibitors, ion channel modulators, enzymes and nuclear receptors^35^.

#### Biological activity spectrum (BAS):

BAS of a best-docked compound represents the complex of pharmacological effects, and the intrinsic properties of the compound depending on its structural property. The pharmacological effects, exhibited by a compound and its communication with biological entities were predicted by PASS online by uploading the SMILES string of natural compounds 36.

## Results and Discussion

### Three dimensional structure prediction:

The predicted model of R. solani Crz1 protein and its threedimensional coordinate files in PDB format were successfully obtained from I-TASSER. The results obtained from a server includes predicted secondary structure with a confidence score (range 0 to 9), predicted solvent accessibility, five predicted structures with C-score, top ten templates from PDB used in alignment, high ten PDB structural analogs, functional analogs protein, and binding site residues. Crz1 Model (Figure 1I) was selected as the best-predicted model with C-score 2.25, TM-score 0.45 ± 0.14, and RMSD 12.1 ± 4.4 Å 18. Top ten threading templates for query protein sequence were identified by LOMETS meta-server (Supplementary Table 1 - available with authors). Normalized Z-score generally estimates the threading alignment. However, a normalized Z-score >1 value reflect a particular arrangement, but in case of small alignment of the large query sequence, it does not give the significant indication of modeling accuracy. The percentage sequence identity in the threading aligned region (Iden1) and the whole chain (Iden2) considered for the first homology (Supplementary Table 1). The structural alignment program, TM-align, identified 5v3jE in PDB library as the best structural analog of the top scoring model of I-TASSER with the TM-score of 0.696 (Supplementary Table 2 - available with authors).

### Assessment of predicted model:

**Figure 2:**
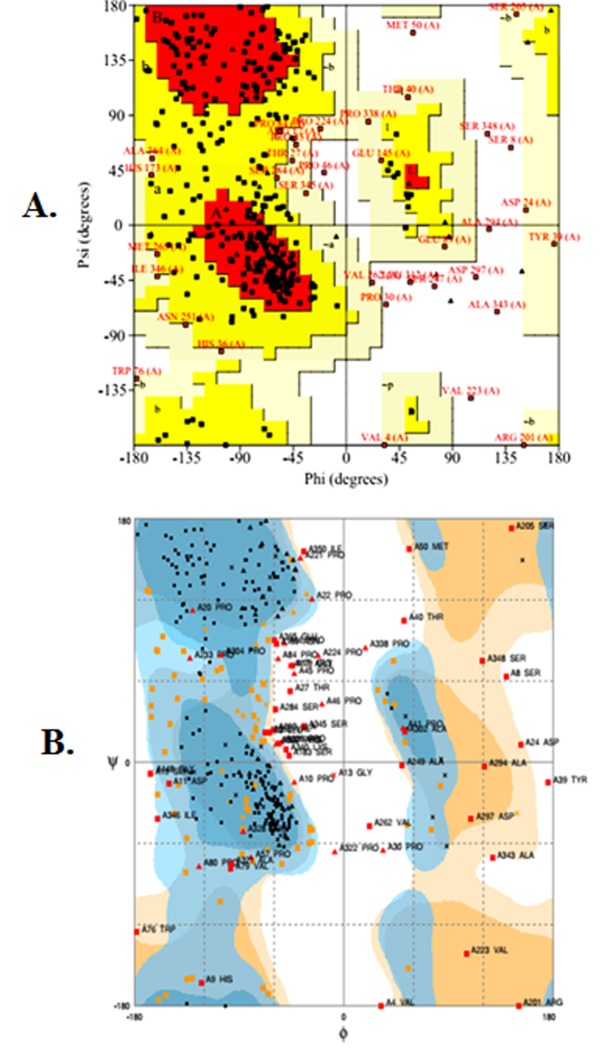
Validation of top score Crz1 model of I-TASSER by (A) PROCHECK Ramachandaran plot (B) Mol Probity Ramachandaran plot.

The Ramachandaran plots of the best-predicted model were obtained from PROCHECK and MolProbity servers which showed the reliability of the model. The PROCHECK Ramachandaran plot showed 89.9% residues in most favored regions and 6.6% residues in additional allowed regions, i.e., the total of 96.5% residues in allowed regions which indicates a good quality model of Crz1 (Figure 2 A). MolProbity Ramachandaran plot also showed 98.0% residues in allowed regions which again confirmed the quality of the predicted model of Crz1 (Figure 2B).

### Active site identification:

**Figure 3:**
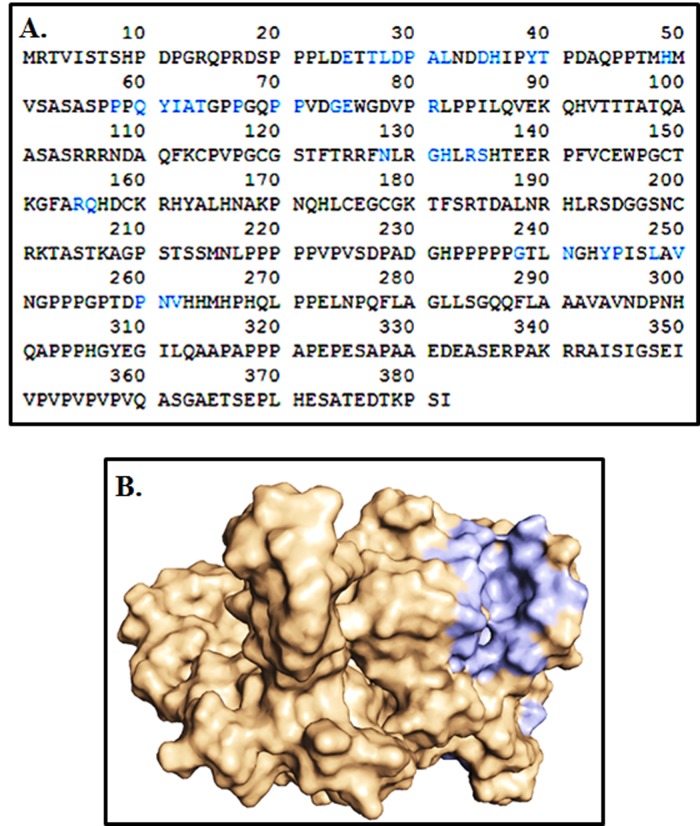
Binding pocket identification by CASTp server: (A) Light blue color boxes highlight the amino acid residues present in the binding site, (B) Shows the binding sites of Crz1 protein.

The protuberant binding site of proteins Crz1 was calculated through the CASTp server with ideal parameters (Figure 3). CASTp evaluation observed the active site amino acids, surface area (3064.847) and volume (10195.168) of Crz1. In ICL1 protein, all 86 binding pockets were categorized to find the residues about probe 1.4 Å radius. The light blue color denotes the active site amino acid residues involved in the formation of binding pockets (Figure 3A and B).

### Docking studies:

Auto docking 4.2 was used to determine the orientation of inhibitors bound in the active site of the Crz1, and the confirmation with the binding energy value for each molecule was chosen for further analysis and results of these studies are given in Table 1. Our efforts towards the development of new Crz1 inhibitors, we investigated the binding modes of Crz1 inhibitors using Discovery studio 3.5 software 29.

**Figure 4:**
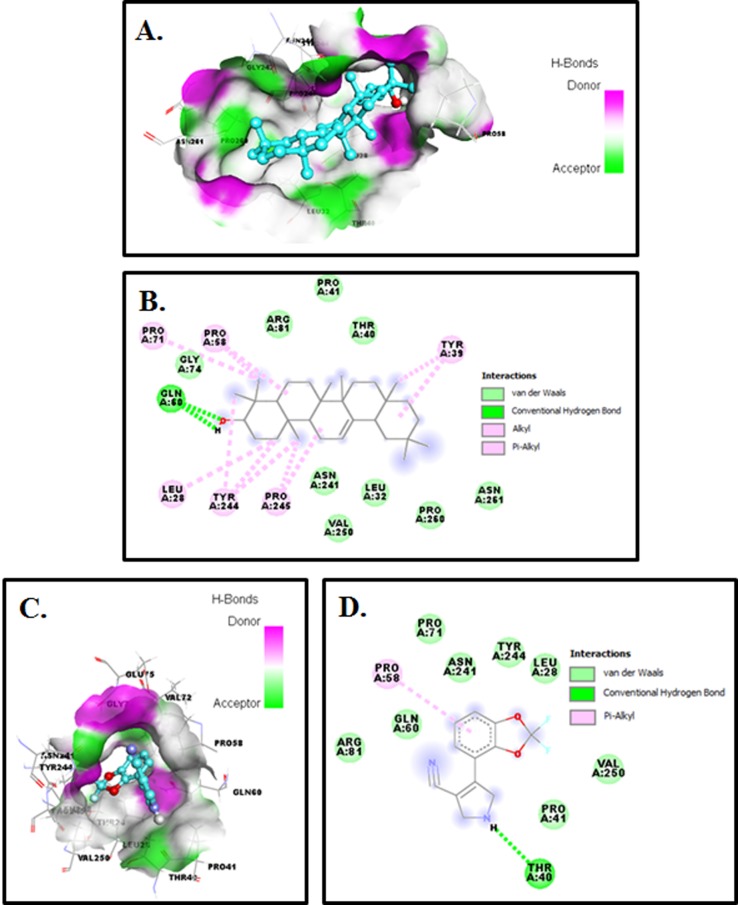
Molecular docking of compounds with Crz1: (A) Surface view of protein with compound Alpha amyrin and (B) 2D schematic diagram showing interactions of compound Alpha amyrin to the Crz1. (C) Surface view of protein with compound Fludioxonil (D) 2D schematic diagram showing interactions of compound Fludioxonil to the Crz1. Residues involved in hydrogen bonding, van der Waals interactions, carbon-hydrogen, and Pi-alkyl are represented in different color indicated in the inset.

Among the 532 natural compounds tested for Crz1 inhibition, Molecular docking response of Crz1 against 532 natural compounds of PubChem database unveiled that compounds 473170, 10494, 222284, 44256718, 5281855, 5280863, 5280343 and 370 yielded the excellent binding energy (ΔG) -9.8, -8.8, -8.4, -8.3, -7.9, - 7.7, -7.7 and -7.7 kcal/mol, respectively compared with reference drugs such as Fludioxonil (Table 1). Protein-ligands relationships profile unveiled that the inhibitors of compounds are found to be mostly reaching to the active site residues of Crz1 like Asp35, His36, His49, Tyr61, Glu25, Thr27, Gln60, Pro260, Asn261, Asn128, Gln156, Pro71, Arg81, Gly74 and associated with hydrogen bonding with bond distance lies within the range of 4 Å (Table 1). Alpha amyrin (73170) was found to be most potent and nicely bounded into the active site of Crz1 with minimum binding energy (deltaG) -9.8 kcal/mol as compared to Fludioxonil (Table 3 and (Figure 4A). Compound 73170 demonstrated two hydrogen bonds with Gln60 of Crz1 at 2.8 Å and 2.4 Å respectively (Figure 4B). The compound 73170 also interacts with the Crz1 binding site by interacting with other residues (Leu28, Leu32, Tyr39, Thr40, Pro41, Pro58, Pro71, Gly74, Arg81, Asn241, Tyr244, Pro245, Val250, Pro260, and Asn261) as compared to Fludioxonil shown in Figure 4B. In Figure 4D, Fludioxonil interacted through two hydrogen bond with Crz1 (Thr40; 3.0 Å and Pro245; 2.4 Å). It communicates with the Crz1 binding site by interacting with other residues (Leu28, Pro41, Pro58, Pro71, Gln60, Arg81, Asn241, Tyr244 and Val250). Molecular docking studies suggested that the numerous van der Waals, covalent, carbon-hydrogen, Pi alkyl, and electrostatic interactions are the critical force for holding of compounds 473170, 10494, 222284, 44256718, 5281855, 5280863, 5280343 and 370 together with the Crz1. Therefore, finally concluded compounds 473170, 10494, 222284, 44256718, 5281855, 5280863, 5280343 and 370 had shown better binding energy for Crz1, and it may be considered as a considerable inhibitor of the Crz1.

### Pharmacokinetics and toxicity:

During clinical trials, most of the drugs flop due to frail pharmacokinetic properties beside with cellular toxicity. Thus, in a silicon profile of pharmacokinetic selected compounds were evaluated for putative bioavailability for Crz1 inhibitors (Table 2). Lipophilicity (clogP), physicochemical properties, polar surface area, molecular weight (MW) and primarily aqueous solubility (logS) are linearly connected to the bioavailability and absorption of drug molecule 37, 38. All these properties are directly affecting the drug movement from its site of regulation to the blood. The CYPs (cytochrome P450) play a significant role in the metabolism of the drug and are evenly notable for nature of drugs in the body, their toxicological and pharmacological effects 39. Here, ADMET (DS3.5) was used to get the predicted profile of pharmacokinetic molecules

It utilizes a model of QSAR to compute the ADMET related properties for small molecules. Along with P value (Lipophilicity) is a significant property for calculating peroral bioavailability of the molecules of the drug. The Results have shown that all basic natural compounds have standard AlogP value ≤ 5. Similarly, all natural compounds showed moderate to a good range of solubility level (solubility level 4 to 3). Only three compounds (5280863, 44256718 and 5280343) showing probability score for hepatotoxicity as ≤ 0.5. The observed value of human intestinal absorption (HIA) is excellent for entire molecules excluding 73170 and 222284. The penetration ability of compounds across blood-brain-barrier (BBB) is high when the prediction value is zero and is least for the prediction value of 4. All natural compounds showed better ability (BBB = 4) 40-45.

The CYP2D6 probability of all natural compounds showed the value of < 0.5 that all compounds were non-inhibitor to CYP2D6 enzyme. For real drug ability, the ideal plasma protein binding (PPB) level is 0. All natural compounds except 5280863, 44256718 and 5280343showed good activity of PPB and came in greater the standard 0. PSA depends upon the confirmation and hydrogen bonding. It shows the single low-energy conformer of the molecule. For the activity of a drug, the optimum value of PSA is ≤ 140 Å 46. The logP and hydrogen bonding value are the two critical descriptors to define the PSA of a drug molecule. All predicted compounds showed significant PSA except 5280863, 44256718 and 5280343.

With the help of the computer-aided toxicity predictor, TOPKAT the cellular toxicity of natural compounds. The carcinogenic and mutagenic effect of compounds with WOE Prediction (weight of evidence) and Ames Prediction was our primary goal. It comprises of various toxicity endpoints and models (irritation, teratogenicity, sensitization, neurotoxicity, and immunotoxicology) that are employed in the development of a drug. All the selected compounds showed Ames probability score ≤ 7 and were non-mutagen. Another toxicity predictor WOE (weight of evidence) was employed to examine the relative level of some compounds that cause cancer in humans. All compounds are predicted as noncarcinogenic except 222284. Therefore, ADMET score and TOPKAT property data of virtual natural compounds have suggested us that the selected molecules might be utilized as bioactive with some minor modification.

### Drug-likeness properties:

Natural compounds were found to possess the best drug-like properties by Lipinski's rule of five shown in Table 3. Interestingly all selected natural compounds bear the Mol. Wt. Range from 170 to 456 (< 500) except Cyanidin 3, five diglucoside and Quercetin. The drug molecules have low, Mol. Wt. (<500) are transported, diffused and absorbed without difficulty in comparison to large molecules. Molecular weight is one of the critical aspects in corrective drug action; if it seems to increase against the absolute limit; the compound size is known to increase correspondingly, which affects the efficiency of the drug. Some hydrogen bond donors (NH and OH) and Number of hydrogen bond acceptors (O and N atoms) are natural compounds as established in Lipinski's limit range from 1- 16 and 1-11 that comes out to be less than 10 and 5 47.

TPSA and the value of Lipophilicity (logP) are the two essential properties in analyzing peroral bioavailability of drug molecule. Permeability possessions of compounds were also studied; the calculated log P value of natural compounds was ranging from 0.12 to 8. 16 (<5). It is known to be the acceptable limit <5 for the drugs to penetrate through bio-membranes 48. Topological Polar Surface Area (TPSA) was calculated from surface areas occupied by oxygen, nitrogen and the hydrogen atoms that are attached to them. Thus, the TPSA is closely related to the hydrogen bonding potential of a compound 49. TPSA has thus known as a perfect descriptor illustrating drug absorption, including intestinal absorption, Caco-2 permeability, BBB penetration, and bioavailability. For the compounds with =ten rotatable bonds and also TPSA of ≤ 140 Å can be said to have Good bioavailability 46.

The molecules are more flexible when the number of rotatable bonds increases and more susceptible to proficient interactions with a precise binding pocket. Exceptionally, all compounds have = ten rotatable bonds and elastic. TPSA of all ligands exhibited 20.23 Å to 140 Å ranges, which indicates good bioavailability by oral route except Kaemferol, Cyanidin 3, 5 glucoside, and Quercetin. Drug likeliness property of natural compounds ion channel modulator, kinase inhibitor, nuclear receptor ligand GPCR ligand, a protease inhibitor, enzyme inhibitory activity and were measured and summarized in Table 4. The bioactivity score of the molecules having more than 0.00 is likely to bear significant biological activities, values -0.50 to 0.00 are presumably active moderately, and when the score comes out to be less than - 0.50, it is supposed as inactive 35. The present study results that the compounds that are investigated were experimented as biologically active and performed the physiological functions by acting together with nuclear receptor ligands, GPCR ligands, inhibit protease and other enzymes. GPCR compounds - the related cascade of signaling were further used for the development of novel functional drugs with better binding specificity profile and less harmful effects. The bioactivity score for GPCR ligand was studied to lie between <0.00 for all tested compounds except cyanidin 3, 5 diglucoside, quercetin, ellagic acid and gallic acid. The ion channel modulator has resulted in the movement of the charged particles across the cell membranes and are valuable targets, that are altered by various drugs having therapeutic value.

The score of bioactivity of ion channel having modulator activity was flanked by >0.00 and -0.50 of all ligands. The results obtained were similar for all compounds, which possess a score value of - 0.50 to 0.00 except Kaemfero that was lying in >0.00. This value shows the Kaemfero can work as Kinase inhibitors. Kinase inhibitors have the property to block or modulate diseased signaling pathways 50. All compounds were found to be in perfect bioactivity scores for the nuclear receptor ligand and inhibition of enzyme as compared to Cyanidin 3, 5 diglucoside, Quercetin, Gallic acid. Interestingly, all compounds were found to be in an excellent bioactive score against, ion channel modulator, GPCR ligand, kinase inhibitor, the protease inhibitor, enzyme inhibitory activity, and nuclear receptor ligand.

### Biological activity predictions:

Using PASS online server, selected bioactive constituents were obtained to evaluate the possible biological activity. The biological activity spectrum (BAS) of a compound is known to have pharmacological effects, specific toxicities, and mechanisms of action occurring due to compounds. Because these probabilities can be calculated independently, the Pa and Pi values vary from 0 to 1, and Pa + Pi < 1. Pa belongs to the class of active whereas Pi is for stable compounds 51.PASS prediction results showed that the highest Pa value than Pi value come off for anti-inflammatory and anti-neoplastic activity and hence indicated the antifungal of selected compounds (evaluated in Table 5). However, all compounds have shown a significant Pa value as compared to Pi value. These compounds might be inhibiting fungal infection via blocking Crz1 action as evidenced by docking studies.

## Conclusion

In conclusion, we have developed PubChem database contain some compounds as possible Crz1 protein inhibitors, by extensive docking experiments validated with biological activity spectrum results. The compounds have shown substantial promising in silico results as reflected by their high binding interaction and considerable high protein-ligand stabilization energy. Analysis of the ADME and QSTR profiles of the selected compounds revealed that these compounds appear to be satisfactory drug liveliness properties. However, molecular docking and biological activity spectrum study are the one way of estimating the activity of the molecules involved. Hence, further research could prove this compound to be a probable anti-fungal drug. With our significant results, all the compounds can further be studied for structural modification, extensive and elaborated investigations to arrive at possibly novel potent agents with little therapeutic activity.

## Conflict of Interest

Authors declare no conflict of interest.

## Figures and Tables

**Table 1:** Binding energy and specific interaction of CRZ1 with compounds

Compounds Name	Pubchem CID	Binding Energy (kcal/mol)	No. of hydrogen bonds	Hydrogen bond forming residues	Distances (�)	Other interacting residues
Compounds Name	Pubchem CID	Binding Energy (kcal/mol)	No. of hydrogen bonds	Hydrogen bond forming residues	Distances (�)	Other interacting residues
Kaemferol	5280863	-7.7	5	Asp35	2.2	Thr27, Leu28, Asp29, Pro30, Ala31, Met50, Ile62, Ala63, Thr64,Pro67, Gly68, Pro71
				His36	2.4, 3.2	
				His49	2.7	
				Tyr61	2.9	
Cyanidin 3,5 diglucoside	44256718	-8.3	11	Glu25	2.4	Thr26, Leu28, Asp29, Pro30, Asp35, Pro45, His49, Gln60, Ile62,Pro70, Pro71, Tyr244, Leu248
				Thr27	2.3, 2.7	
				His36	2.7	
				Tyr61	2.3, 2.5	
Quercetin	5280343	-7.7	5	Gln60	2.9	Leu32, Tyr39, Thr40, Pro41, Pro58, Pro59, Pro70, Arg81, Gly238, Thr239, Asn241, Pro245, Val250, Val262
				Pro260	2.6	
				Asn261	2.3, 2.7, 2.1	
Alpha amyrin	73170	-9.8	2	Gln60	2.8, 2.4	Leu28, Leu32, Tyr39, Thr40, Pro41, Pro58, Pro71, Gly74, Arg81, Asn241, Tyr244, Pro245, Val250, Pro260, Asn261
						
Oleanolic acid	10494	-8.8	---	---	---	Thr26, Thr27, Leu28, Ala31, His36, Gln60, Tyr61, Ile62, Ale63, Thr64, Pro66, Pro67, Gly68, Pro70, Pro71
Beta sitosterol	222284	-8.4	1	Asn261	2.2	Leu28, Leu32, Tyr39, Thr40, Pro58, Gln60, Pro71, Gly74, Arg81, Asn241, Tyr244, Pro245, Val250, Pro260, Val262
Ellagic acid	5281855	-7.9	2	Asn128	3.3	Phe123, Gly131, His132, Arg134, Ser135, Ala154, Arg155, His157
				Gln156	2.9	
Gallic acid	370	-7.7	4	Pro71	2.3, 3.4	Thr26, Leu28, Pro58, Gln60, Val72, Glu75, Asn241, Tyr244, Pro245, Leu248, Val250
				Arg81	3.5	
				Gly74	3.1	
Known Inhibitors						
Fludioxonil	86398	-6.5	2	Thr40	3	Leu28, Pro41, Pro58, Pro71, Gln60, Arg81, Asn241, Tyr244, Val250
				Pro245	2.4	

**Table 2:** Pharmacokinetics profile of natural compounds

Pubchem ID	Compounds	BBB	AlogP	Sol.	HIA	HTL	HT_Prob	PPB	CYP2D6	PSA	Ames Mut.	Prob	Enrichment	WOE
5280863	kaemferol	0	2.872	3	0	0	0.11	0	0	190.12	NM	0.11	0.2	C
44256718	Cyanidin 3,5 diglucoside	0	2.872	3	0	0	0.36	0	0	260.61	NM	0.29	0.53	NC
5280343	Quercetin	0	3.688	3	0	0	0.11	0	0	265.43	NM	0.26	0.46	NC
73170	a-Amyrin	4	7.303	0	3	1	0.71	2	0	20.81	NM	0	0	NC
10494	Oleanolic acid	2	1.345	4	0	1	0.92	2	0	41.46	NM	0.7	1.27	NC
222284	�-Sitosterol	4	8.084	0	3	1	0.54	2	0	20.81	NM	0	0	C
5281855	Ellagic acid	4	1.584	3	1	1	0.97	1	0	135.72	NM	0.32	0.57	NC
370	Gallic acid	3	0.733	4	0	1	0.6	1	0	100.56	NM	0.58	1.05	NC

**Table 3:** Molecular properties of natural compounds

	Compounds	Milogp	TPSA	No. of atoms	MW	H-bond Acceptor	H-bond Donor	Volume	nrotb
	Kaemferol	0.12	190.28	32	448.38	11	7	364.19	4
	Cyanidin 3,5 diglucoside	-4.61	270.61	43	611.53	16	11	499.05	7
	Quercetin	-1.06	269.43	43	610.52	16	10	496.07	6
	a-Amyrin	8.02	20.23	31	426.73	1	1	460.7	0
	Oleanolic acid	6.72	57.53	33	456.71	3	2	471.14	1
	�-Sitosterol	8.62	20.23	30	414.72	1	1	456.52	6
	Ellagic acid	0.94	141.33	22	302.19	8	4	221.78	0
	Gallic acid	0.59	97.98	12	170.12	5	4	135.1	1

**Table 4:** Bioactivity scores of compounds

	Compounds	GPCR	ICM	KI	NRL	PI	EI
	Kaemferol	0.05	-0.05	0.1	0.2	-0.05	0.41
	Cyanidin 3,5 diglucoside	-0.06	-0.49	-0.21	-0.17	-0.06	-0.06
	Quercetin	-0.05	-0.52	-0.14	-0.23	-0.07	0.12
	a-Amyrin	0.22	-0.05	-0.31	0.67	0.11	0.56
	Oleanolic acid	0.28	-0.05	-0.4	0.77	0.15	0.65
	�-Sitosterol	0.14	0.05	-0.51	0.73	0.07	0.51
	Ellagic acid	-0.29	-0.27	-0.01	0.11	-0.18	0.17
	Gallic acid	-0.77	-0.26	-0.88	-0.52	-0.94	-0.17

**Table 5:** Biological activity spectrum of compounds (Pa � Active; Pi � Inactive)

	Name of the compounds	Pa	Pi	Activity
	Kaemferol	0.719	0.019	Antifungal
	Cyanidin 3,5 diglucoside	0.642	0.012	Antifungal
	Quercetin	0.711	0.018	Antifungal
	a-Amyrin	0.449	0.066	Antifungal
	Oleanolic acid	0.848	0.02	Antifungal
	�-Sitosterol	0.851	0.01	Antifungal
	Ellagic acid	0.698	0.042	Antifungal
	Gallic acid	0.848	0.033	Antifungal
